# The Optimal Position of the Implant Supporting Distal Extension Removable Denture: Case Reports and Literature Review

**DOI:** 10.7759/cureus.71284

**Published:** 2024-10-11

**Authors:** Hassnae Benyahia, Jihane Slaoui, Imad Al-Banyahyati, Meryem Rhissassi, Salwa Berrada

**Affiliations:** 1 Prosthodontics, Faculty of Dental Medicine, Mohammed V University, Rabat, MAR; 2 Prosthodontics, Faculty of Dentistry, Mohammed V University, Rabat, MAR; 3 Prosthodontics, Abulcasis International University of Health Sciences, Rabat, MAR; 4 Periodontology, Faculty of Dental Medicine, Mohammed V University, Rabat, MAR

**Keywords:** distal edentulism, implant, implant supported removable prosthesis, patient’s satisfaction, removable partial denture, strategic position

## Abstract

The management of distal extension edentulism by removable partial denture (RPD), in cases where fixed solutions cannot be considered, poses various challenges inherent in the mixed support of the prosthesis, especially when the edentulism is of a large extent. When an implant is placed to assist a removable denture, it allows ensuring a bio-functional, aesthetic, and comfortable rehabilitation. Additionally, it renders possible resolutions to problems related to conventional RPD. The implant can be placed in proximity or at a distance from abutment teeth, with each position presenting its own advantages and benefits. The choice of the optimal position is made following a clinical examination and cone beam computed tomography. Through two clinical cases, we will illustrate the management of distal edentulism with implant-assisted RPD, then review the key elements to consider during the prosthetic realization in the management of extended edentulism and discuss the parameters involved in the choice of preferred implant sites.

## Introduction

The number of partially edentulous patients is constantly increasing; the choice of treatment plan depends on several variables, such as the patient’s oral and systemic health, preference, compliance, available budget, and the clinician’s knowledge and therapeutic skills. Literature reveals that many patients, especially the elderly, refuse to receive an implant-supported fixed prosthesis, even in the absence of any systemic pathologies contraindicating implant rehabilitation, usually citing reasons such as cost, fear, refusal of certain surgical procedures, or satisfaction with their current removable denture [1]. In many cases, the conventional removable partial denture (RPD) is a prosthetic solution that satisfies the expectations and functional requirements of our patients. However, it presents almost constant aesthetic, biological, and biomechanical disadvantages. The association of implants as a complementary means of retention offers undeniable advantages and benefits to the RPD [2]. The placement of an implant allows the elimination of the vestibular clasp arm, which causes aesthetic discomfort; the optimization of the stability, especially in the distal extension medium and large edentulous; the preservation of the osteomucosal support structures by limiting the resorption [3]; and the protection of the periodontal structures, especially those bordering the edentulism. The masticatory efficiency and the satisfaction index are then clearly improved compared to a conventional RPD [4]. After planning, the practitioner chooses the position of the implant, which can be placed anteriorly or correspond to the molar sites, each position having its own benefits. The choice of position must allow the prosthesis to benefit from all the advantages that the association of the implant can provide with a minimum of interventions. The success and survival rate of the rehabilitation depends mainly on the therapeutic planning that guides the optimal placement of the implant, the design of the metal framework, and compliance with the right prosthetic steps. Through two clinical cases, we will focus on implant-assisted RPD, discuss the parameters involved in the choice of preferred implant sites, and review the key elements in the prosthetic management of the distal extension edentulous.

## Case presentation

First case presentation

**Figure 1:**
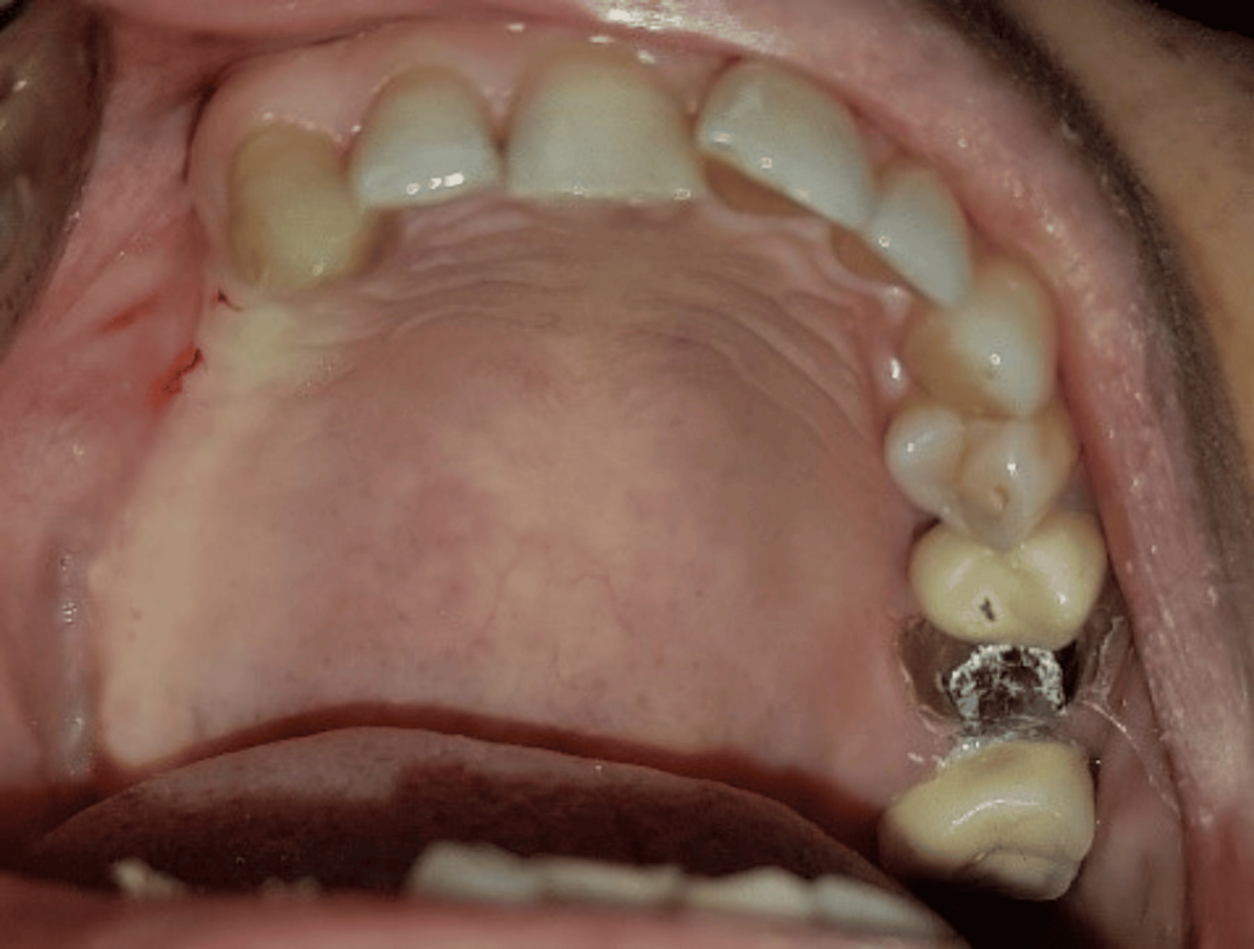
Intra-oral view of the patient.

A 58-year-old female patient was referred to the Department of Prosthodontics for Maxillary Prosthetic Rehabilitation; the patient did not have any specific medical history. The patient had a gummy smile; the intra-oral examination revealed a maxillary Kennedy's class II extended to 13 and a narrow ridge (Figure 1). She never had any previous rehabilitation. With a Class II Division 2 malocclusion, dental malposition, and anterior cervical asymmetries, the patient expressed that these particular anomalies posed no concern for her.

**Figure 2:**
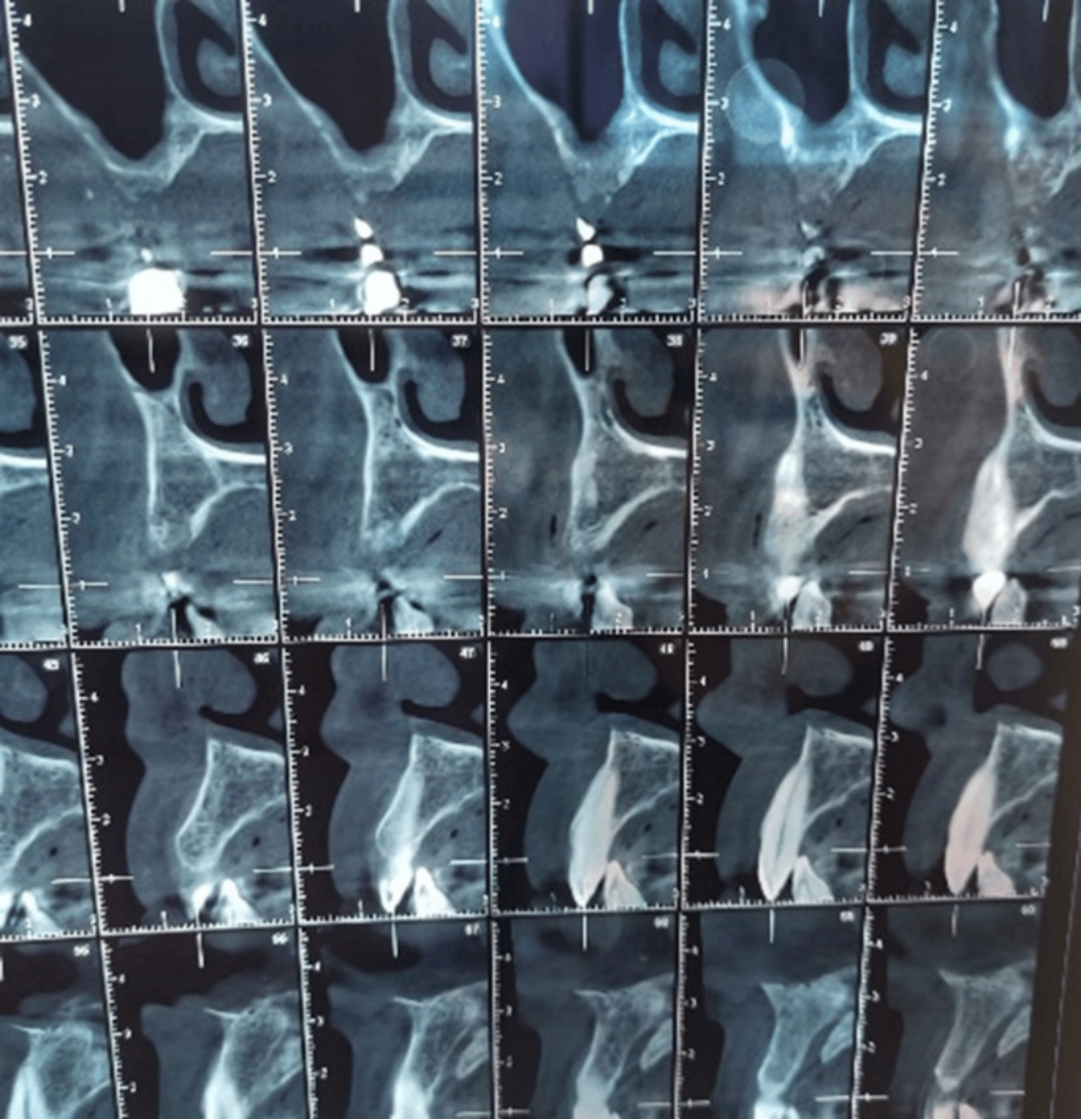
CBCT evaluation. An oblique coronal CBCT view showing a low bone height except at the site of the 14.

Clinical examination and cone beam computed tomography (CBCT) revealed an atrophy of the maxilla due to sinus pneumatization, resulting in reduced bone volume (Figure 2). However, a favorable site for implantation was located at the site of the 14. After clinical assessment and CBCT analysis, various prosthetic solutions were proposed to the patient: a bridge supported by three implants with sinus augmentation at sites 16, 17, and 14, a stabilized RPD anchored at the site of 14, and a conventional RPD with a clasp on the 13. The patient refused the surgical procedures necessary for an implant-supported fixed prosthesis. Consequently, the decision was made to opt for a removable partial denture stabilized by an implant located at the site of the first premolar.

Surgical management

**Figure 3:**
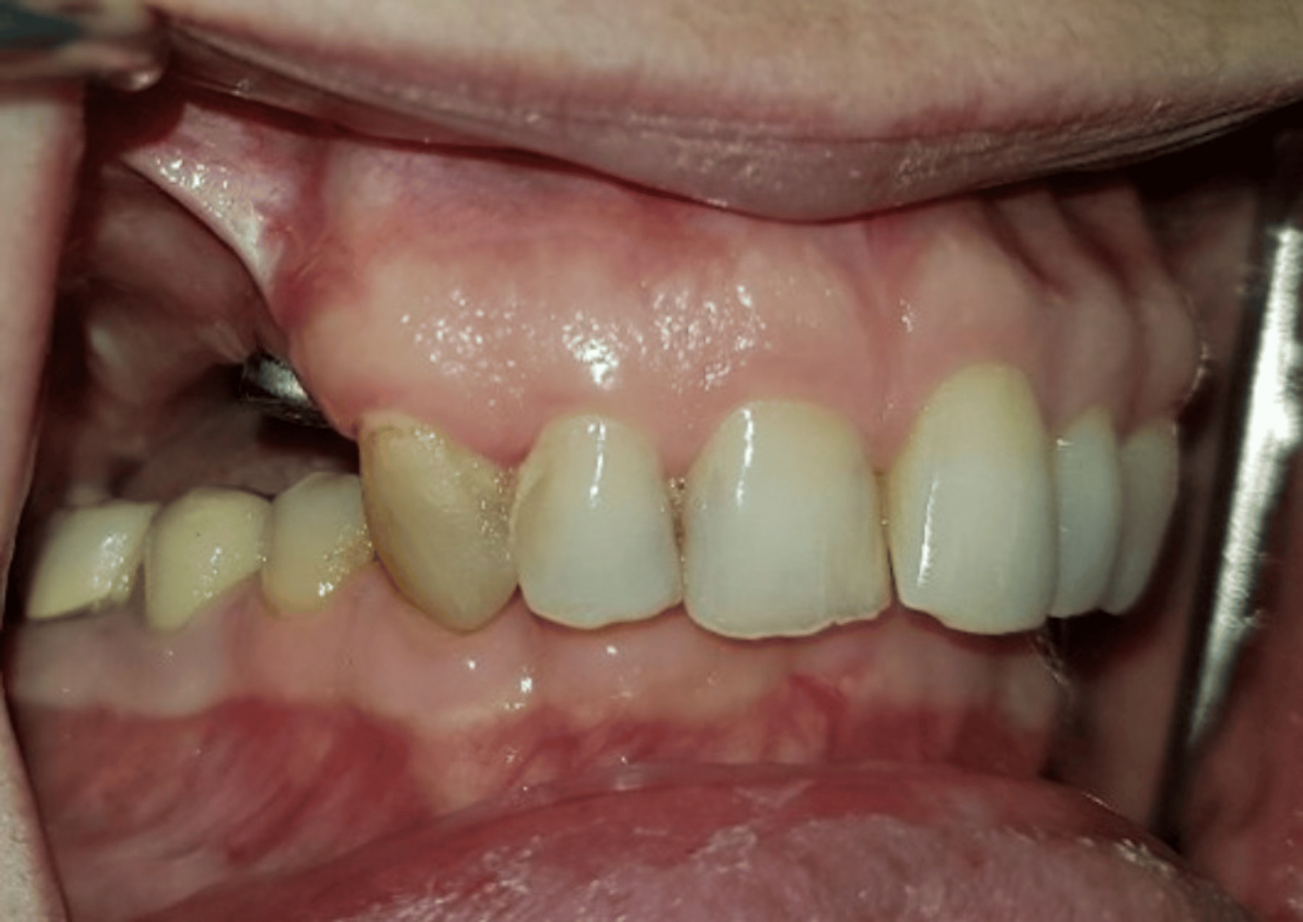
Implant placed at the site of the first premolar.

The implant is placed at the site of 14 using a surgical guide, originally employed as a radiological guide, and identified as the sole favorable site for implantation without prior preparation. Considering the unfavorable bone density and the poor primary stability, the healing screw was placed after two months during a second stage (Figure 3).

Prosthetic management

**Figure 4:**
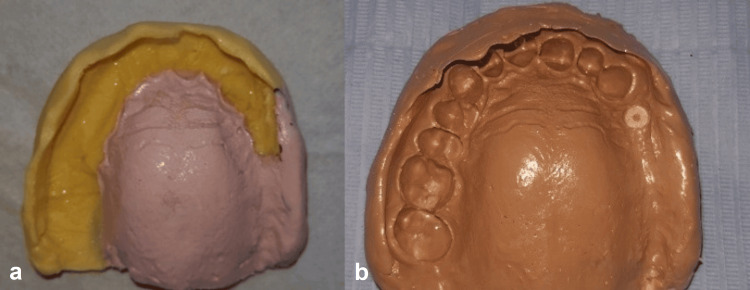
Secondary impression. a: Initial stabilization impression made with zinc oxyphosphate. b: Global impression made with polysulfide medium and light viscosity.

After coronoplasties, a global impression was taken with a custom tray and border molding, using materials with different viscosities (Figures 4a, 4b).

**Figure 5:**
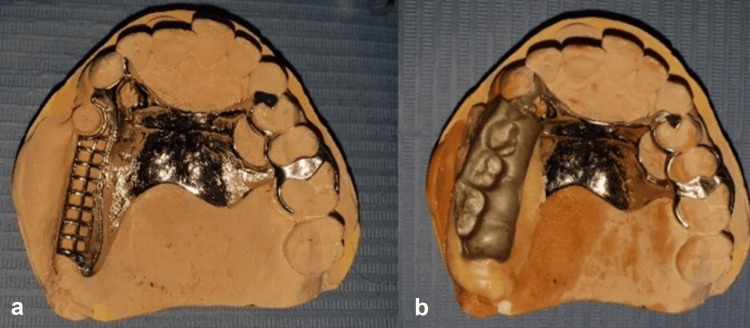
Maxillo-mandibular record. a: Framework design. b: Recording precision of the maxillo-mandibular with reinforced wax on top of the resin seat cover.

The framework design adhered to standard rules, with the only modification being the removal of the buccal clasp's arm on the 13 (Figure 5a). The maxillo-mandibular relationship was recorded using the framework, which, once coated with resin, acted as a base record occlusion. The recording was refined with reinforced wax (Figure 5b).

**Figure 6:**
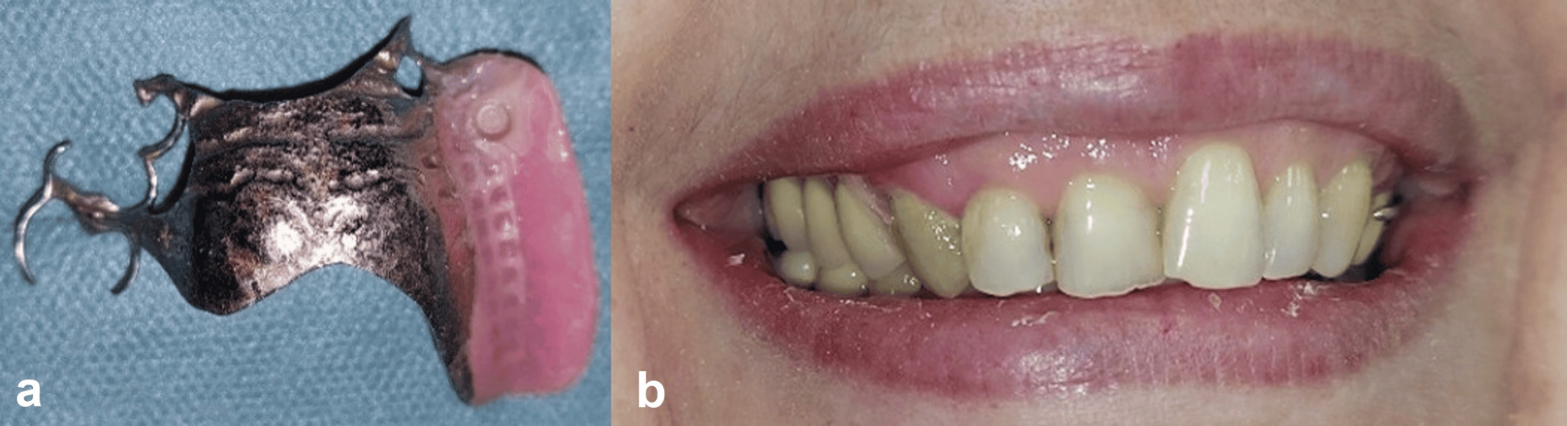
Female part solidarization. a: Metallic female part incorporated in the prosthetic intaglio. b: Prosthesis inserted, resulting in a satisfied smile.

Due to the reduction of prosthetic volume buccolingually, the metallic female part could have caused metal visibility through transparency and an elevated risk of resin facing breakage around the implant; the retentive plastic insert is thus inserted directly in contact with the resin (Figure 6a). After four years, the patient remains very satisfied both functionally and aesthetically. There have been no reported issues or complaints during this period (Figure 6b).

Second case presentation

A 75-year-old woman was sent to the prosthodontics department by her referring dentist for the renewal of her unstable RPD after a fracture of the canine supporting a single clasp. The patient had no notable medical history and expressed a desire to preserve her remaining maxillary teeth. On extraoral examination, she had a medium smile line with the presence of the four maxillary anterior incisors only, restored by a bridge with an acceptable aesthetic result. The edentulous ridges had a clinically satisfactory volume. The patient did not express dissatisfaction with the removability of her old prosthesis, which had been functional for several years. The CBCT revealed sufficient bone volume in width and height at all evaluated sites. Since the patient presented with maxillary subtotal edentulism, the proposed prosthetic solutions included: an implant-supported fixed prosthesis requiring four implants per quadrant for restoration of the right and left sectors; an RPD stabilized by two implants for each sector; an RPD stabilized by one implant for each sector located anterior to the edentulous area; and a RPD stabilized on an implant located at the molar site for each sector.

The patient, accustomed to removability and limited resources, opted for the last proposed option of an RPD stabilized by an implant located in each sector. The chosen site was the first molar position.

Surgical management

The placement of the implants at the appropriate site was possible thanks to the surgical guide used for the cone beam. The healing screws were placed during the first surgical stage, considering the good primary stability obtained during the placement.

Prosthetic management

**Figure 7:**
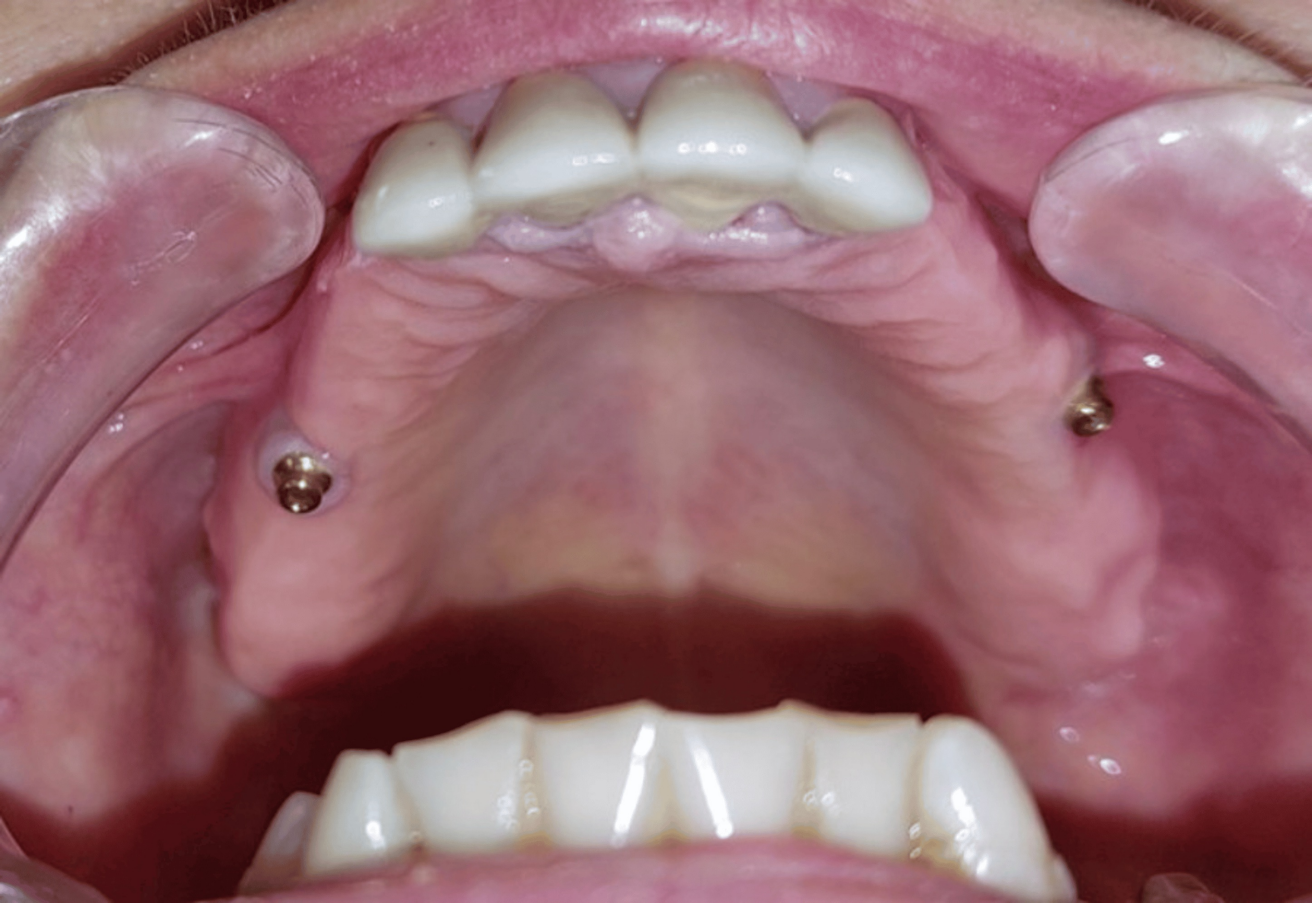
Male part of the attachment that emerges from 1 mm (locator attachment).

The attachment was chosen after three weeks, the period required for the healing of the mucosal sleeve; the measurement of its height by a graduated probe allowed the choice of the male part of the attachment, which should emerge by 1 mm to 2 mm (Figure 7).

**Figure 8:**
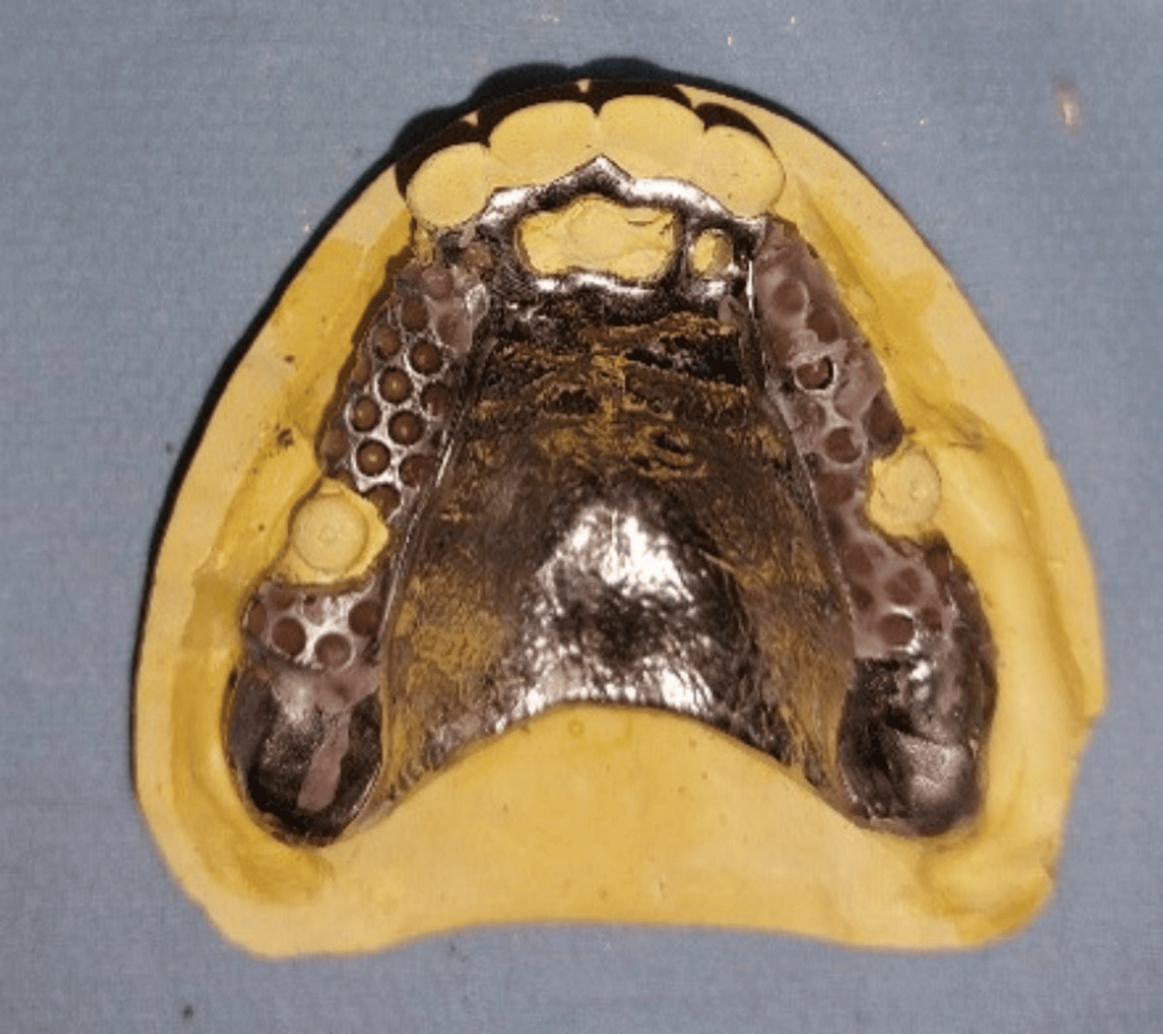
Metal framework that complies with the biomechanical requirements.

The design of the framework complies with standard biomechanical requirements; only the buccal clasp’s arm on the anterior teeth is eliminated (Figure 8). An anatomo-functional impression was performed to appreciate the difference in depressibility between the various structures supporting the RPD, using an impression tray remargined with thermoplastic paste and taken in two successive steps with elastic materials with different viscosities. The solidarization was made directly after placing the spacer and the female part with the plastic insert. The prosthetic intrados is hollowed out, and a fluid resin is placed to ensure proper retention of the female part, which is then fixed.

**Figure 9:**
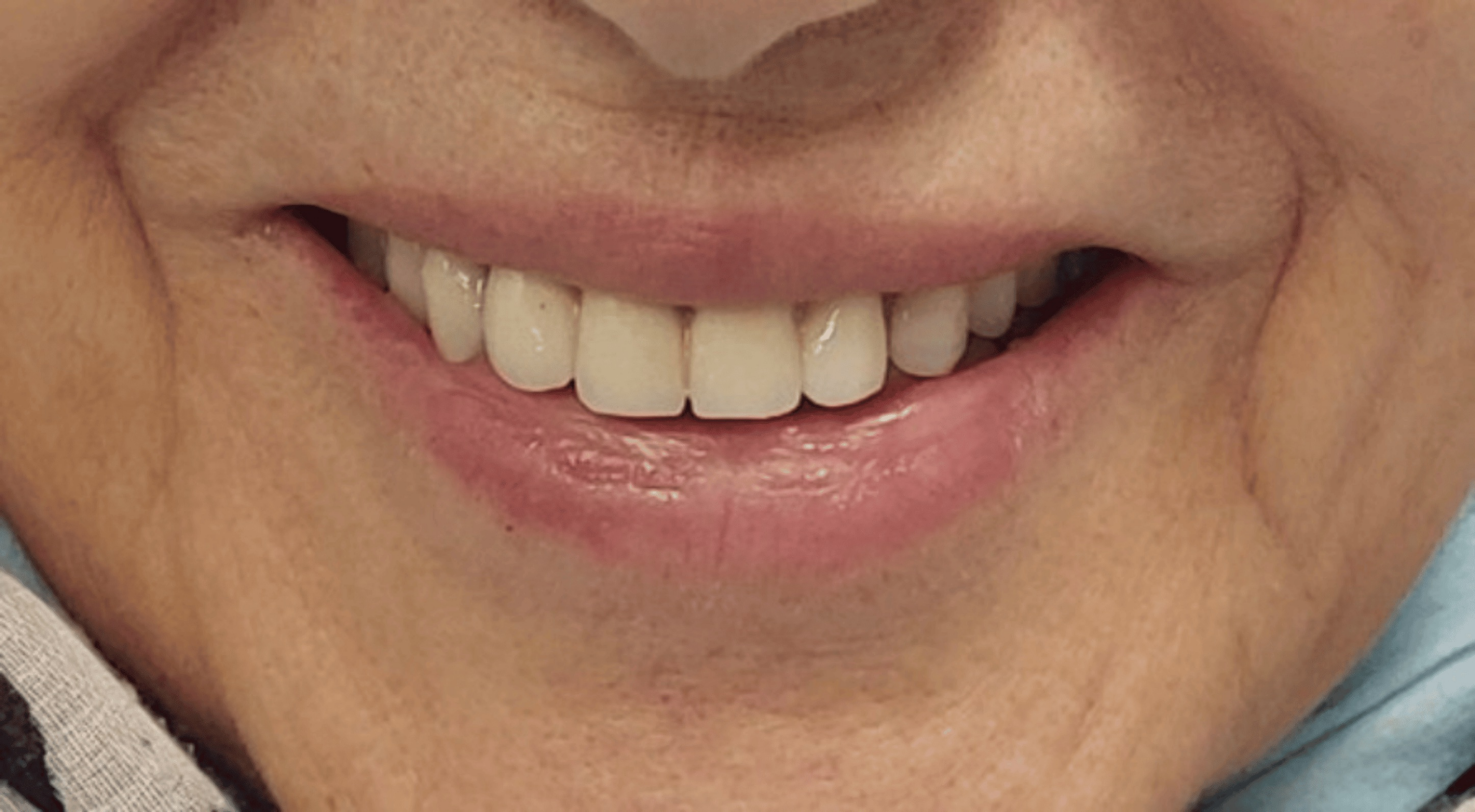
Patient’s satisfied smile after solidarization of the attachment.

The patient was immediately pleased with the aesthetic result of the prosthesis, which was natural and functionally integrated (Figure 9). The patient is entirely satisfied three years after the prosthetic insertion.

## Discussion

The stabilized RPD is a removable prosthesis with a metal framework associated with one or two implants supporting a superstructure that plays the role of an additional retention element. This chosen prosthetic solution addressed the aesthetic demand in the first case related to the inevitable appearance of the clasp in a conventional RPD. It also helped avoid multiple interventions and the relatively high cost associated with a fixed solution. In the second case, the implant-stabilized RPD allowed the preservation of the patient's last remaining teeth since a conventional solution could not have been considered, considering the reduced number of remaining teeth with low retentive values, while a fixed implant-supported solution would require a high number of implants, a significant cost, and would not meet the patient’s request as she was accustomed to the removability of her prosthesis for several years [5].

In the first case, a standard implant was placed near the tooth bordering the edentulous area, the only site favorable to implantation without prior preparation. In the second case, a standard implant was placed in the first molar site for stabilization of the RPD. In distal extension edentulism, the most described situation is the posterior position, which offers greater stability and considerably limits bone resorption [6]. Its position at the level of the first molar is preferable according to several studies because it decreases the stress on the remaining teeth, improves the stability of the prosthesis in the vertical plane, and optimizes the masticatory efficiency as the maximal occlusal forces are exerted in this region [7,8]. When the implant is placed in the third molar position, it can cause significant stress on the terminal teeth bordering the edentulous area. However, this position will improve the support and stability of the prosthesis since the distal edentulism is transformed into a bounded edentulism. However, when the anatomical conditions do not allow the placement of an implant at the posterior level, its placement anteriorly or adjacent to the abutment tooth is imagined and presents its own advantages; in fact, it helps reduce the occlusal force on the abutment and shows satisfying results [9-11].

Considering the dental-osteo-mucosal supports of the implant-support RPD, one or two implants per quadrant are sufficient to ensure an optimal balance of the RPD; they can be standard, short, or narrow [12,13]. Nevertheless, the number will depend on the clinical context: the extent of edentulism, the nature of the antagonist arch, the type of implant used, and the number of favorable sites for implantation. Moreover, regarding the exact position of the implant, it does not systematically correspond to the center of the future prosthetic tooth unless a fixed rehabilitation is considered by the patient later on since the use of the implant as a means of stabilization of the RPD does not compromise its survival rate if the planning is carefully established [14]. Implants are ideally placed parallel to the insertion axis of the RPD in order to minimize the concentrated stress on the implant and anticipate the accelerated deterioration of the prosthetic components, particularly the plastic inserts, to be minimized [15]. However, if a slight discrepancy exists, its recovery is possible thanks to specific resilient inserts. With regard to the attachment used, there is insufficient evidence that there is a significant difference between the various attachments in terms of retention, prosthetic support, and implant survival rate [16]. The implant survival rate depends on the forces transmitted by the prosthesis. These forces must comply with the biomechanical requirements of the prosthesis design. This ensures that loads are distributed over all the support structures rather than concentrated solely on the implant. Signs of overloading will be noted regardless of its position.

## Conclusions

The molar position of the implant has the advantage of reducing resorption and ensuring optimal balance of the prosthesis. The anteriorly placed implant reduces stress on the abutment tooth and preserves its proximal bone. Whichever position is chosen, the advantages are undeniable and effective; the choice of position will depend essentially on the position of the site favorable to implantation. The RPD stabilized on implant is a therapeutic option that offers the patient an aesthetic, functional, and comfortable rehabilitation in the management of his distal edentulous, compared to fixed solutions that would require additional costs and steps that are not easily accepted by all patients.
